# Comparison of T-cell receptor repertoire restriction in blood and tumor tissue of colorectal cancer patients

**DOI:** 10.1186/1479-5876-8-35

**Published:** 2010-04-12

**Authors:** Sebastian Ochsenreither, Alberto Fusi, Susanne Wojtke, Antonia Busse, Natascha C Nüssler, Eckhard Thiel, Ulrich Keilholz, Dirk Nagorsen

**Affiliations:** 1 Charité, Campus Benjamin Franklin, Department of Hematology and Oncology, Hindenburgdamm 30, 12200 Berlin, Germany; 2 Department of General and Visceral Surgery, Klinikum Neuperlach, Städtisches Klinikum München, Oskar-Maria-Graf Ring 51, 81737 Munich, Germany; 3 University of Heidelberg, Medizinische Fakultät Mannheim, Department of Hematology/Oncology, Theodor-Kutzer-Ufer 1, 68167 Mannheim, Germany; 4 Micromet AG, Staffelseestr 2, 81477 Munich, Germany

## Abstract

Several immunotherapeutic approaches rely on antigen-specific T-cells. Restrictions in the T-cell receptor (TCR) repertoire were reported as indicator of anti-tumor cytotoxic T-lymphocyte (CTL) response in various tumor entities. It is unclear yet whether a TCR restriction in peripheral blood mirrors the tumor compartment. We compared the expression of TCR *Vβ*-families for the quantification of TCR repertoire alterations in blood and tissue samples from patients with colorectal carcinoma. Blood samples from patients with colorectal carcinoma and healthy volunteers and tissue samples of normal colonic mucosa and colorectal carcinoma were analyzed. Relative *Vβ*-family quantification was performed based on quantitative reverse transcribed PCR. Standard deviation and average mean of the single families were determined. Two variables describing the degree of *Vβ*-repertoire restriction were defined. Forty-eight blood samples and 37 tissue samples were analyzed. TCR repertoire restriction was higher in blood of tumor patients than in blood of healthy controls (*p *< 0.05). No difference in the degree of TCR repertoire restriction was found between carcinoma and unaffected colon tissue. We found no corresponding elevated TCR families among the different compartments blood, normal colon, and carcinoma tissue of the same patient. In conclusion, we observed a repertoire restriction in peripheral blood as well as in tumor tissue of cancer patients. However, in tumor tissue, repertoire alterations were comparable to normal mucosa, suggesting compartment-specific TCR distribution rather than alterations due to tumor-T-cell interaction questioning the presence of highly restricted clonal T-cell expansions in colorectal cancer as they have been described in other, assumingly more immunogenic tumor entities.

## Background

Understanding the interaction between tumor and immune system might help improving immunotherapeutic approaches for malignant diseases. T-cells directed against tumor associated antigens (TAA) could play a key role in the surveillance of and in the defense against tumor cells [1]. In fact, spontaneous T-cell responses against TAAs have been described in peripheral blood, lymph nodes, and bone marrow of patients with various malignant diseases prior to immunotherapy [2].

In colorectal cancer (CRC), spontaneous T-cell responses against several TAAs have been detected in peripheral blood, particularly in patients with metastatic disease [3,4]. No evidence was found that these spontaneous, peripheral TAA-specific T-cells have an impact on survival of CRC patients [5]. Therefore, the focus of interest has moved to tumor-infiltrating T cells. CD8+ T-cell infiltration of CRC is known to be associated with a better prognosis, but it is still unknown whether these infiltrating T cells, in fact, represent expanded tumor specific T-cell clones [6-13].

In case of unknown or multiple epitopes, the analysis of TCR repertoire both by FACS and PCR based methods offers the opportunity to detect oligoclonal expansion of specific T-cells [14-16]. The dimeric transmembrane T-cell receptor (TCR) is the central mediator of epitope specific cytotoxic T-cell activation. Consisting of an *α*- and a *β*-chain in most of the cases, diversity is generated during T-cell evolution by recombinations of the gene segments *V *(*variable*), in case of the *β*-chain *D *(*diversity*), and *J *(*joining*) to a constant chain gene *C *[17]. *V*-genes are grouped in families consisting of genes with sequence homology of at least 50% [18]. For analysis of the TCR repertoire, the *β*-chain is often preferred because of the lower number of families even if a higher overall variability of sequence compared to the *α*-chain has been described [19]. Alterations in TCR repertoire can be evaluated either by length or sequence analysis of the highly variable part of the *α*- or *β*-chain for each *V*-family [14,20-22] or by quantification of the single families by southern blot, FACS, or quantitative reverse transcribed PCR (qRT PCR) [23-27].

In cancer research, a restricted TCR repertoire has been found at the tumor site of various malignant diseases [28-36], and in case of melanoma, a highly restricted repertoire may be linked to regression during cytokine therapy [37]. However, it is still a matter of debate whether a restricted TCR repertoire in peripheral blood of tumor patients exists and whether such a peripheral restriction mirrows oligoclonal expansions of specific T-cells in the tumor compartment [36,38-42].

We used a qRT PCR-based relative *Vβ*-family quantification approach [27] for analysis of TCR *Vβ*-family expression. Especially in the gut, lymphocytes bearing γδ TCR are abundant, which are potentially involved in an antitumoral response in an MHC-independent manner [43]. Assessing *Vβ*-family restriction, clonal expansions of γδ T-cells are not addressed. Aim of the study was the application of mathematical markers to describe the global restriction of the αβ TCR repertoire in the different compartments rather than the detection of single expanded T-cell clones. From this general point of view we evaluated whether or not significant differences of TCR repertoire restriction can be detected in samples from carcinoma patients and healthy controls as well as in tumor tissue compared to unaffected colonic mucosa.

## Materials and methods

### Specimen collection

Peripheral blood samples were drawn from patients and healthy volunteers. Tissue samples both of carcinoma and unaffected mucosal tissue were collected from patients affected by CRC undergoing tumor resection. RNA was extracted from the macroscopic center of the tumor and from unaffected colonic mucosa at least 5 cm from the macroscopic border of the malignant lesion. Age, sex, and in CRC patients TNM and UICC stages were assessed. Both patients and controls had given informed consent for the use of their specimens before sampling.

### RNA extraction, cDNA synthesis

Total RNA was extracted from peripheral blood mononuclear cells (PBMCs) or fresh tissue using TRIzol^® ^(Invitrogen, Carlsbad, CA, USA) or RNeasy^® ^Mini Kit (Qiagen, Hilden, Germany). Reverse transcription was performed with Omniscript Reverse Transcriptase^® ^(Qiagen) as described previously [44]. Samples were stored at -20°C.

### Quantification of TCR expression

For determination of general TCR expression, the *Cα*-chain was quantified on a LightCycler^® ^instrument (Roche, Basel, Switzerland). Values were normalized using the low-abundance housekeeping gene porphobilinogen deaminidase (PBGD) as previously described [27]. For each cDNA synthesis reaction (+RT), a control reaction without reverse transcriptase was performed (-RT control). Samples with -RT/+RT ratio >0.1 for *Cα*-chain or PBGD indicating DNA contamination and/or RNA degradation were excluded from further analysis. The aim of our study was the detection of TCR repertoire restriction due to T-cell expansions associated with significant TCR expression of the expanded clones. Low overall TCR transcription in tissue could lead to putative oligoclonality due to the generally high sensitivity of PCR based approaches. Consequently, samples with HAC/PBGD < 0.1 were excluded from analysis to avoid this bias.

### Relative quantification of Vβ-families

Relative quantification of the expression of a single TCR *Vβ*-chain was performed as described [27]. Shortly, qRT PCRs were performed with a universal reverse primer and TaqMan probe, both annealing at the constant part of the *β*-chain (*Cβ*), and 28 *Vβ*-family-specific forward primers (*Vβ*-family 1 to 24, *Vβ*-families 5, 6, 12, 13 subdivided into two subgroups each). Slope of each family specific reaction was estimated analyzing a dilution series spanning three orders of magnitude of a cDNA mixture of diagnostic samples. Calculation of the relative concentration *P*_*j *_[%] of a *Vβ*-family *j *was carried out using the formula

with *Cp [amplification cycles] *is the Crossing point and *[ΔCp/log(concentration)] *is the average slope of all families.

### Normalization, quantification of Vβ-restriction

**Figure 1:**
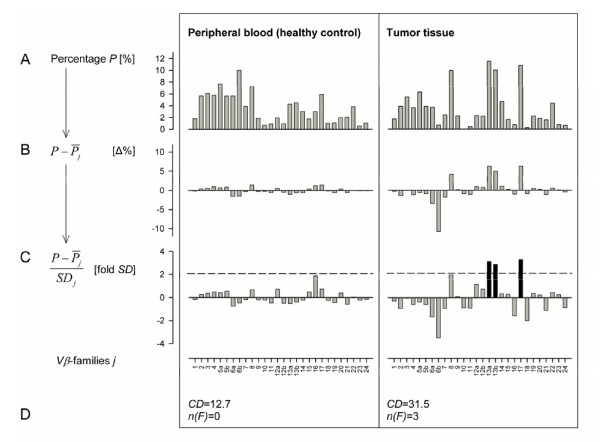
**Normalization of the relative concentrations *P*_*j*_**. Two exemplary samples are shown (PBMCs from peripheral blood of a healthy control: left, carcinoma tissue: right). Shown are the single steps of normalization: relative concentration *P*_*j *_(A), *P*_*j *_minus the average value of Family *j *of all analyzed samples (B), and deviation from average given in fold of standard deviation of the respective family (C). The sum of the modulus' normalized concentrations of all families of the samples (*CD*) and the number of families elevated more than average plus two SD *n(F) *are indicated at the bottom (D)

The approach calculating relative concentrations of different *Vβ*-families regarding slopes and crossing points, as a matter of fact, leads to per-sample normalized values. A per-family normalization step was added to circumvent different weighting of family alterations due to different physiological family expressions and PCR amplification efficacies. For this purpose, the mean percentage  and standard deviation *SD*_*j *_of every *Vβ*-family *j *of all analyzed samples *k *were determined. It was decided not only to use PBMCs from blood of healthy donors but all analyzed samples for normalization leading to more reliable results comparing repertoire restriction of tissue with blood. Per-family normalized relative concentration *P'*_*jk *_of a family *j *of sample *k *was calculated as follows:

For estimation of the general degree of alteration in the *Vβ*-family repertoire, we defined a variable *CD *('cumulative deviation'). For each family *j *of a sample *k*, the value of deviation from the mean percentage of all analyzed samples  was expressed as multitude of *SD*_*j *_of this family. *CD*_*k *_of a sample was defined as sum of the moduli of these normalized deviations:

To accommodate the fact, that in tumor immunological settings, a mono- or oligoclonal T-cell response is postulated [16], which is supposed to be associated with the elevation of only a single or very few increased families, we defined a second marker *n(F)*. For each sample *k*, the number *n(F) *of families was determined, which were expressed higher than the mean percentage  plus two standard deviations *SD*_*j *_(*P*'_*j *_> 2). In contrast to *CD *depicting the sum of expression deviations from the average of all *Vβ*-families, only significantly elevated families were relevant for the value of the second marker *n(F)*. The applied normalization procedure is depicted exemplary in Figure 1.

### Statistical methods

All statistical tests were performed as two-tailed tests and a *p*-value < 0.05 was considered significant. Correlations were tested by calculation of the Pearson coefficient. Mann-Whitney U test was applied to unpaired samples, Wilcoxon test was used for paired samples.

## Results

### Patients and specimens

Fifty-one samples from peripheral blood (21 samples of healthy controls and 30 samples from carcinoma patients) and 58 tissue specimens both from normal colon tissue of CRC patients and colon carcinoma tissue were analyzed. Fourteen samples (three from peripheral blood, five samples of unaffected colon, and six samples originating from carcinoma tissue) had -RT/+RT ratios > 0.1. Six tissue samples had a HAC/PBGD ratio < 0.1 corresponding to minimal TCR expression (one sample of unaffected colon, five samples of tumor tissue). Altogether 19 Samples fulfilling one or both precondition were excluded from further analysis. Altogether, 48 blood and 42 tissue samples were evaluable. In total, samples of 19 healthy donors (peripheral blood) and 40 CRC patients (peripheral blood: n = 29, healthy colon: n = 24, carcinoma: n = 18) were analyzed. Characteristics of patients and healthy controls are depicted in Table 1.

**Table 1:** Characteristics of patients and healthy controls

	Healthy controls (n = 19)	CRC patients (n = 40)
Age (years)		
Range	40-63	43-90
Median	54	70

Gender (n)		
Male	4	25
Female	15	15

UICC stage (n)		
n. d.	n.a.	4
1		4
2		16
3		10
4		6

n. d.: not determined

n.a. not applicable

To test the reliability of our normalization approach, we calculated the theoretically expected percentage of samples with at least one family elevated more than two SD (*n(F) *>0) and compared that value with results from all samples analyzed. Assuming a symmetric Gaussian distribution of relative concentrations within a family, the expected percentage of samples with *n(F) *>0 would be 49%. Of all 90 samples analyzed, 46 (51%) showed *n(F) *>0 demonstrating high reliability of our method. Families with frequently low expression were tested negative more often than others (negative correlation between average relative concentrations  and the number of negative PCRs of a family *j *in all evaluable samples, *R*_*p *_= -0.4286, *p *= 0.0229). However, after normalization, the absolute number a family *j *was considered as elevated (*n(F) *>0) in all evaluable samples was independent of the average relative expression  of that family (data not shown).

### TCR Cα expression in blood and tissue specimens

**Figure 2:**
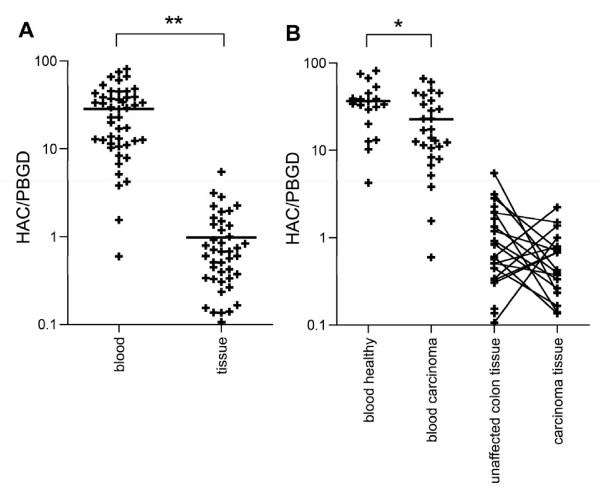
**Relative *C*α concentrations (HAC/PBGD) of the analyzed samples**. (A) HAC/PBGD was significantly higher in blood samples (healthy controls and carcinoma patients) compared to tissue samples (unaffected colon and carcinoma tissue, ** *p *< 0.0001). (B) HAC/PBGD was higher in blood samples of healthy controls than in samples of carcinoma patients (* *p *= 0.0235, unpaired). No difference in HAC/PBGD was detected comparing tissue samples of unaffected colon with samples of carcinoma tissue (*p *= 0.1147, paired).

As expected, *Cα *(HAC/PBGD) was expressed significantly higher in peripheral blood than in tissue (*p *< 0.0001). HAC/PBGD was higher in blood samples of healthy volunteers compared to carcinoma patients; but no difference in *Cα*-expression was found between unaffected colon and tumor tissue in a paired analysis (Figure 2). These results have to be interpreted with caution because of the fact that HAC/PBGD was used as criteria to select samples for further repertoire analysis. Without excluding samples with HAC/PBGD < 0.1, *Cα *concentration was lower in the carcinoma samples than in samples of unaffected mucosa (paired, *p *= 0.047).

**Figure 3:**
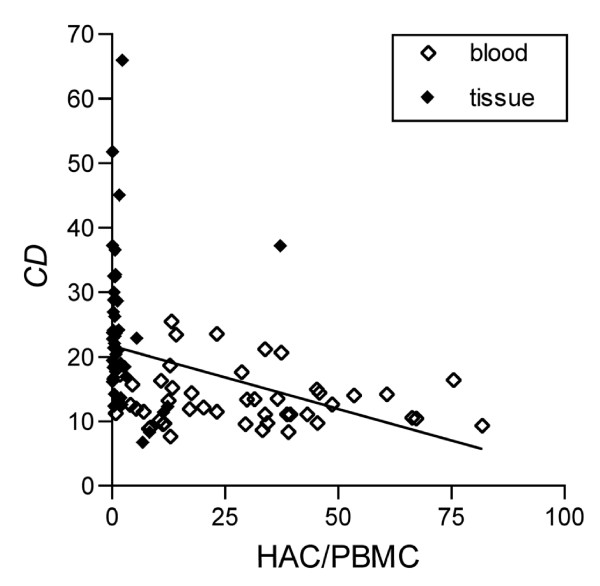
**Correlation between relative *C*α expression (HAC/PBGD) and cumulative deviation *CD***. According to the higher HAC/PBGD expression in blood samples compared to tissue specimens, we found a strong negative correlation between HAC/PBGD and *CD *analyzing all specimens irrespective of origin (regression line, *R*_*p *_= -0.4004, *p *= 0.0003). Regarding samples from blood (empty diamonds) and tissue (filled diamonds) separately, no correlations between the mentioned parameters were determined (*R*_*p*, *blood *_= -0.1108, *p*_*blood *_= 0.4585, *R*_*p*, *tissue *_= 0.01344, *p*_*tissue *_= 0.9286).

According to the differences of expression between blood and tissue specimens, analyzing all evaluable samples irrespective of origin, we found a negative correlation between HAC/PBGD and *CD *(Figure 3) as well as HAC/PBGD and *n(F) *(not shown). However, separate analysis of the samples originating from blood and originating from tissue samples resulted in no significant correlations (Figure 3 and not shown).

### Vβ repertoire restriction in peripheral blood

TCR repertoire analysis of PBMCs from 19 healthy donors and 29 colon carcinoma patients showed in 16 out of 48 samples one or more families with *P*_*j*_' >2 (2 out of 19 samples from healthy donors (11%), 14 out of 29 samples from cancer patients (48%)) reflecting an non-normalized expression of one or more families higher than the mean percentage  plus two standard deviations *SD*_*j *_(*n(F) *>0). In the statistical analysis, both *CD *and *n(F) *were higher in PBMCs from cancer patients than in PBMCs from healthy volunteers (unpaired, *p*_*CD *_= 0.0316, *p*_*n*(*F*) _= 0.0195, Figure 2B).

### Vβ repertoire in tissue samples of CRC patients

**Figure 4:**
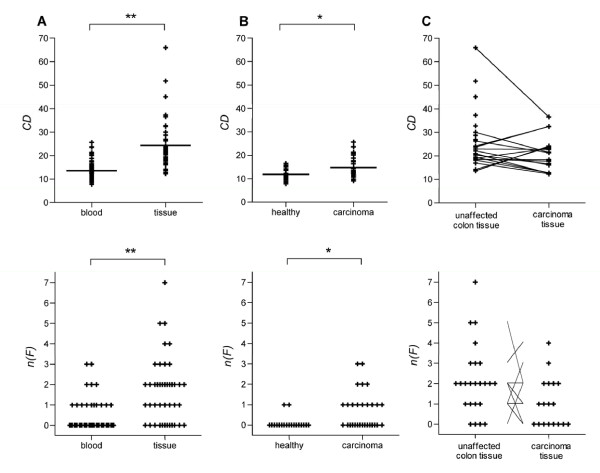
**Statistical comparison of repertoire restriction degree measured in *CD *and *n(F)***. Compared were samples from blood with tissue (both normal colon and carcinoma, A), blood from healthy controls with blood from carcinoma patients (B), and tumor free colon with carcinoma samples both originating from CRC patients (C). * p < 0.05, ** p < 0.01.

Twenty-four samples of normal colon tissue and 18 carcinoma samples were evaluable. The statistical analysis of both *CD *and *n(F) *comparing all tissue samples (carcinoma and unaffected colon) with all blood samples (healthy controls and carcinoma patients) showed a significantly higher repertoire restriction in tissue than in blood (unpaired, *p*_*CD *_< 0.001, *p*_*n*(*F*) _< 0.001, Figure 4A). However, no difference was observed between samples of unaffected colon and carcinoma tissue (paired, *p*_*CD *_= 0.471, *p*_*n*(*F*) _= 0.2783, Figure 4C).

### Comparison of elevated families in different compartments

For 16 patients, samples of tumor and tumor free colon were available. In the carcinoma samples, altogether 30 families were elevated (0-5 families per patient). In the corresponding samples of unaffected colon tissue, 14 families were elevated (0-4 families per patient). In only two of these patients a single family was elevated in both tumor tissue and corresponding unaffected colon. For nine CRC patients, blood and tumor samples were available. Four of these patients had elevated families in peripheral blood, but none of these families was elevated in the corresponding tumor sample. Please refer to figure 4.

## Discussion

In this study, TCR *Vβ*-family repertoire restrictions in blood and tissue of patients with colorectal carcinoma were compared using high throughput relative quantification of TCR *Vβ*-families based on qRT PCR technology. While a multitude of mathematical approaches have been established to describe the degree of repertoire restriction in single V-families from spectratype/immunoscope data (reviewed in [45]), to our knowledge, a model to describe global TCR repertoire restriction based on V-family quantification has not been published so far.

Colorectal carcinoma is thought to be of limited immunogenicity. Nevertheless, global *Vβ*-repertoire restriction degree in blood reflected by the *CD *and *n(F) *values was significantly higher in carcinoma patients confirming the results of former studies investigating *Vβ*-repertoire restrictions in blood of patients with other tumor entities, which have been interpreted as indication for the induction of specific clones reactive to autologous tumor [26,42]. In fact, this TCR repertoire restriction in tumor patients might be attributed to cumulative *Vβ*-alterations caused by discrete epitope-specific T-cell expansions triggered by several different antigens. Because of the epitope-independency of the assay, unspecific antigen confrontation due to barrier disruption as postulated in the context of other tumor entities [28,32] causing additional repertoire alterations can not be excluded. We observed a difference of *Cα*-chain expression between healthy controls and carcinoma patients, which could have impact on the TCR *Vβ *restriction grade. It remains elusive whether or not this difference is a result of the malignant disease or the higher median age of the patient cohort compared to the healthy control group.

Corresponding significant TCR *Vβ*-family elevations in blood and tumor tissue were not found in the present study. From previous analyses in colorectal cancer, we know, that TAA-specific T-cells circulated with frequencies lower than 1% of CD8+ cells without proven clonality [3]. As anticipated, the proportion of peripheral TAA-specific T-cells was too low to be detected by TCR *Vβ*-quantification due to physiological variation of the relative *Vβ*-family expressions. Assuming several TAAs, expansions of T-cell clones would affect various *Vβ*-families additionally reducing sensitivity of the relative *Vβ*-quantification regarding single family percentages. It is purely speculative to assume that such a specific T cell clone might be trapped at the tumor site and is therefore, not detectable in the peripheral blood.

In carcinoma tissues, we did not observe an elevated repertoire restriction compared to corresponding tissue of unaffected colon. Infiltration of CTLs in colon carcinoma tissue had been identified as prognostic factor suggesting TAA associated CTL activation [12]. Interestingly, we found no difference in the expression of *Cα *comparing healthy colon and carcinoma tissue. These inconsistent observations could be explained by recent results of Salama et al., who showed, that CD8+ CTL density is reduced but CD4+/CD25+/FoxP3+ T regulator cells are elevated in colon cancer compared to normal colon tissue [46,47]. The fact that clonal CTL expansions are not principally associated with restrictions in *Vβ*-family usage of the T regulatory cell population of the same compartment [42,47] might explain the absence of TCR restriction differences between healthy colon and CRC tissue. Independently of these considerations, we have to conclude that the present molecular quantitative TCR analysis is not suitable for the identification of local expansions in colorectal cancer tissue (if they exist) compared to normal colon tissue. This may differ in other compartments and/or for other tumor entities.

Regarding *p*-values comparing the different sample groups (blood of healthy controls, blood of carcinoma patients, unaffected colonic mucosa, carcinoma tissue) one has to keep in mind the differences in sample sizes and applied tests. The highly significant difference in *CD *between blood and tissue samples but the absence of a significant difference between unaffected colon and tumor tissue does not necessarily mean hat there would be no difference to detect if we would compare the same sample size of tissue samples as we used for comparing tissue with blood samples (48 vs 42 instead of 24 vs 18). Regarding the repertoire restriction in blood (healthy vs carcinoma patients) and tissue (unaffected colon mucosa vs carcinoma tissue), the results of the two statistical tests cannot directly be compared because of differences in distribution, range, and dependency of the data sets. However comparing tumor tissue to unaffected mucosa, we observed an even lower median value in carcinoma tissue. Consequently, the absence of a significantly higher *Vβ *restriction in tumor tissue must not be attributed to the slightly smaller sample size compared to the blood samples.

Taken together, we observed an increased TCR repertoire restriction in blood of colorectal carcinoma patients compared to blood of healthy controls using qRT-PCR based *Vβ*-family quantification. A similar degree of TCR restriction in colorectal carcinoma tissue in comparison to normal colon tissue contrasts with the phenomenon of high proliferative oligoclonal expansions of specific T-cell populations as described for highly immunogenic malignancies such as melanoma [16].

## Competing interests

The authors declare that they have no competing interests.

## Authors' contributions

SO conceived of the study, developed and supervised the molecular analyses carried out in this trial, performed the statistical analysis and drafted the manuscript.

AF carried out molecular measurements and analyses, and statistical analyses.

SW collected samples and carried out molecular measurements.

AB carried out molecular measurements and analyses.

NCN collected samples and participated in the conduct of the study.

ET participated in design and conduct of the study.

UK participated in design and conduct of the study.

DN conceived of the study, coordinated the study and drafted the manuscript.

All authors have read and approved the final manuscript.
